# The alternate role of direct and environmental transmission in fungal infectious disease in wildlife: threats for biodiversity conservation

**DOI:** 10.1038/srep10368

**Published:** 2015-05-20

**Authors:** Farah N. Al-Shorbaji, Rodolphe E. Gozlan, Benjamin Roche, J. Robert Britton, Demetra Andreou

**Affiliations:** 1 Bournemouth University, Fern Barrow, Talbot Campus, Poole, Dorset, BH12 5BB, UK; 2 UMR BOREA IRD-MNHN-Université Pierre et Marie Curie, Muséum National d’Histoire Naturelle, 47 rue Cuvier, 75231 Paris cedex 5, France; 3 International Research Unit UMMISCO (IRD/UPMC 209), Center for Mathematical and Computational Modeling of Complex Systems, 32 Avenue Henri Varagnat, 93143 Bondy Cedex, France

## Abstract

Emerging fungal pathogens have substantial consequences for infected hosts, as revealed by the global decline of amphibian species from the chytrid fungus. According to the “curse of the Pharaoh” hypothesis, free-living infectious stages typical of fungal pathogens lengthen the timespan of transmission. Free-living infectious stages whose lifespan exceeds the infection time of their hosts are not constrained by virulence, enabling them to persist at high levels and continue transmitting to further sensitive hosts. Using the only Mesomycetozoea fungal species that can be cultured, *Sphaerothecum destruens*, we obtained tractable data on infectivity and pathogen life cycle for the first time. Here, based on the outcomes of a set of infectious trials and combined with an epidemiological model, we show a high level of dependence on direct transmission in crowded, confined environments and establish that incubation rate and length of infection dictate the epidemic dynamics of fungal disease. The spread of Mesomycetozoea in the wild raise ecological concerns for a range of susceptible species including birds, amphibians and mammals. Our results shed light on the risks associated with farming conditions and highlight the additional risk posed by invasive species that are highly abundant and can act as infectious reservoir hosts.

Generalist pathogens are increasingly causing significant worldwide declines in a wide range of host species leading to considerable changes in their communities^1^,^2^,^3^. As generalist pathogens can transmit to multiple hosts, their virulence is less evolutionarily constrained than specialist disease agents^4^. In the absence of what would otherwise be strong selective pressures, their outbreaks in the environment often lead to significant losses of species, especially if the pathogen has a free-living stage that can survive outside its hosts in the environment^5^,^6^,^7^,^8^.

Free-living infectious stages (such as spores and zoospores) lengthen the timespan of transmission for a pathogen, enabling contact with greater numbers of susceptible hosts^9^. This is consistent with the “curse of the Pharaoh” or “sit and wait” hypothesis, which predicts that free-living infectious stages whose lifespan exceeds the infection time of their hosts are not constrained by virulence, enabling them to persist at high levels of virulence and continue transmitting to further sensitive hosts^10^,^11^,^12^,^13^,^14^. Furthermore, where high proportions of susceptible hosts are present in a community, resulting high mortality rates are associated with significant modifications to trophic structure and ecosystem function^15^,^16^. These modifications include species dependent on depleted populations that struggle to survive, or tolerant hosts thriving in the new community as they can transmit the infectious agent without any clinical signs of disease or mortality.

Typically, emerging fungal pathogens are generalists, especially in humid and aquatic habitats^16^,^17^,^18^,^19^. They have recently been responsible for infectious outbreaks worldwide that have led to high biodiversity and economic costs^6^,^16^,^20^,^21^. A newly emerged group, the Mesomycetozoea, on the animal-fungal boundary includes species that can infect and cause mortality in mammals, fish, birds and amphibians, and has led to noticeable infectious threats to wildlife^22^,^23^. Due to the aquatic nature of their hosts, mesomycetozoea are very difficult to monitor in the wild^24^ which makes it extremely difficult to obtain reliable data on patterns of transmission and virulence. They are also difficult to culture in the laboratory with only one exception, *Sphaerothecum destruens*^25^,^26^ which has allowed tractable data on infectivity and life cycle to be obtained for the first time^27^,^28^.

Here, combining a unique set of infectivity trials and a custom epidemiological model, we aim to characterise the most influential epidemiological parameters in a fungal pathogen’s outbreak using *S. destruens* as a model system. Specifically, we examine the role of alternate transmission methods (direct and environmental) in disease outbreaks and the effect of infection and incubation duration. These parameters are central to the epidemiological understanding of disease dynamics and control.

## Materials and Methods

### Ethics statement

All animal procedures followed strict guidelines set forward by the Home Office, UK and were performed in accordance with UK Home Office Regulations. The project was approved by the Bournemouth University ethics committee and was performed under the Home Office project licence number PPL80/1979.

### Model species

**Figure 1:**
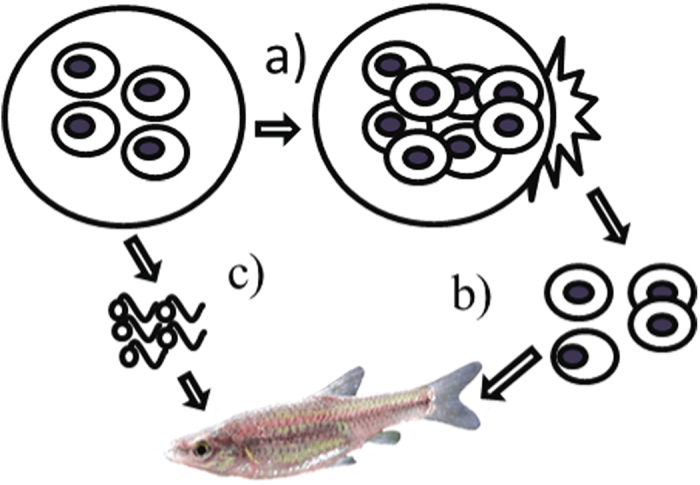
Lifecycle of *Sphaerothecum destruens* .**a**) Spores multiply within host cells until cell death; **b**) Spores spread within the host and are released into the water through urine, bile, or gut epithelium; **c**) In freshwater, each spore can divide into up to 5 uniflagellate zoospores and survive for several days depending on the water temperature. Infection occurs directly or indirectly by ingesting the spores, attachment to the gills or skin, or gut penetration. Photo R. E. Gozlan

*Sphaerothecum destruens* is a fungal pathogen that multiplies within host cells, resulting in a slow-growing infection in various organs and leading to chronic mortalities in the wild. The spores are transmitted to other organs and into the water through bile, urine, and intestinal epithelium, where in freshwater they undergo zoosporulation and can be transmitted to other individuals (Fig. 1)^29^. *S. destruens* is also carried by the highly invasive fish species *Pseudorasbora parva* which shows a high tolerance to the parasite and acts as a disease reservoir^30^. See Supplementary Materials (Table S1) for known *S. destruens* hosts.

### Infectivity trials

Three fish hosts of socio-economic importance, common bream *Abramis brama*, roach *Rutilus rutilus* and common carp *Cyprinus carpio*, were challenged with *S. destruens* infections^31^. One-year old juveniles of each fish species were housed separately in three replicate tanks of 20 individuals each. All fish were exposed to infectious *S. destruens* spores at three separate occasions by bath immersion. Fish were placed in special exposure aquariums where they were exposed to a spore concentration of 8.6 × 10^4^ spores ml^−1^ for 4 hours. At the end of the exposure all fish were returned to spore-free aquaria. Infection could have occurred at the time of exposure or subsequently due to direct and indirect transmission. Mortalities were recorded daily for11 months post exposure with the resulting mortalities in the exposed groups : 53% in *A. brama*, 37% in *R. rutilus*, and 8% in *C. carpio*. Infection by *S. destruens* was confirmed using PCR detection methods in fish tissue samples and histological examination of the fish tissues.

In a separate experiment another susceptible cyprinid, sunbleak *Leucaspius delineatus* was exposed to *S. destruens* via intraperitoneal injection as well as bath immersion in two separate exposure trials for 84 days^26^. We used these combined datasets to calibrate and define the parameters and initial conditions of the model, with the aim of creating a model that can be used across all exposure scenarios.

### Susceptible-exposed-infectious-recovered model (SEIR)

**Figure 2:**
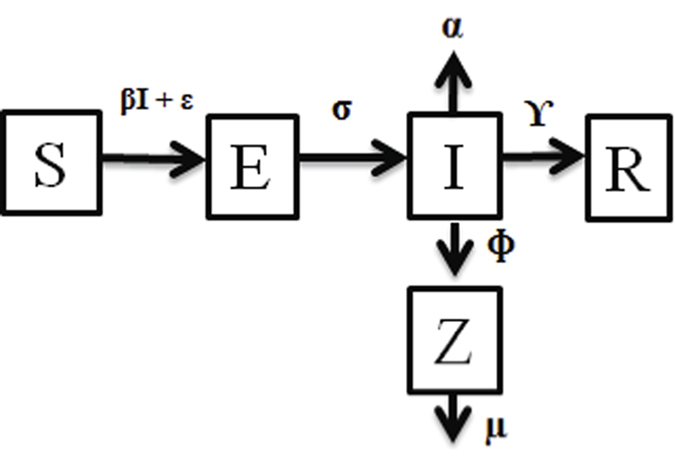
A visual representation of the SEIR model categories. Susceptible individuals (S) are exposed to infection at rate β (direct transmission) + ε (environmental transmission). In the exposed state (E), individuals become infectious (I) at rate σ. Infectious individuals either die as a result of disease at rate α, or recover (R) at rate °. Infected individuals release spores (Z) at rate f, including zoosporulation, which have a collective mortality rate of μ.

We modelled the pathogen-host systems using an adaptation of the SIR framework^32^ (Fig. 2). To calibrate the model more accurately to the observed data, we assumed a closed system which disregards natural birth and death rates of the population. Mortality that occurred in the control tanks, where hosts were sham exposed to the pathogen was classified as natural mortality, and factored out of the experimental mortalities before analysis. A series of differential equations determined the rate of change of the population between compartments corresponding to stages of infection. Susceptible individuals (S) become infected (Eq.1) through direct transmission such as contact (β) with infectious individuals (I) as well as through environmental transmission from the water column (ε) through spores or zoospores (Z):
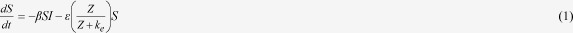


The role of spores and zoospores in this host-parasite system is unquestionably important, as infection occurs through contact with and ingestion of these spores. The constant k_e_ is the number of spores required for a 50% probability of infection, as has been done for other pathogens such as *Vibrio cholera*^33^ or avian influenza viruses^34^. For these systems, epidemiological parameters such as recovery rate are not dose dependent, as hosts have a fixed contact rate with the environment. However, in our study system hosts are constantly exposed to the environmental free-living stages of the pathogen (spores and zoospores) which can potentially alter some important processes of the epidemiological system. A higher level of spores in the water may accelerate the progress of infection, as spores accumulate in the host more rapidly. Thus, this can accelerate the incubation, mortality and recovery rates. Moreover, this assumption makes sense considering that this group of pathogens has shown a dose dependent disease progression^35^, and the necessity of this assumption was tested for each host species.

Epidemiological models on such systems are very rare. Thus, we fitted our epidemiological model to experimental data by considering both the presence and absence of dose dependent disease progression for each epidemiological parameter following infection. For datasets that use bath immersion as an infection method, elevated spore levels lead to the saturation functions behaving as linear functions. Thus, the addition of the saturation function does not help or harm the model fit. However, for a host infected via intraperitoneal injection with low initial free-living spore concentration, the saturation functions improved the model fit over models that did not include the saturation functions (Table 1). For that reason, we include saturation functions for all the parameters, to allow a good fit across all species and all experimental datasets. Our results demonstrate that dose dependent disease progression is important for fitting our model to the experimental data (see Supplementary materials for additional details).

Thus becoming infectious, recovered or dead is correlated to the concentration of spores and zoospores in the environment, as a dose-dependent progression of infection is assumed. The threshold values k_s_, k_g_, and k_a_ were introduced for σ, °, and α respectively to reflect this dose-dependent response and better represent the slow growing and chronic nature of the infection in the wild^36^. Exposed (E) individuals (Eq.2) are infected but not yet infectious (they are not releasing spores) and became infectious (I) after an incubation period of 1/σ*(Z/Z + k_s_) days:
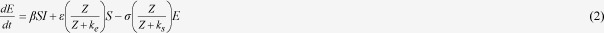


Infectious individuals either experienced mortality as a result of disease at rate α*(Z/Z + k_a_), or can recover (R) at rate °*(Z/Z + k_g_), corresponding to the length of infection (Eq. 3 and 4):
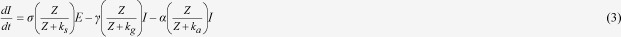

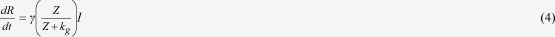


We assumed that recovered individuals are not re-infected. As a result of the experimental design, we assumed that all individuals came into contact with the pathogen. We also assume that individuals that did not die either overcame the infection without noticeable signs of deterioration or were naturally immune. After the experiments were complete, hosts were dissected and tissue samples tested for *S. destruens* presence. However, it must be noted that it is impossible to differentiate between recovered hosts and naturally immune hosts at the end of the experiment.

Spores released into the water from infected individuals can divide into up to 5 zoospores (additively, they constitute Z) at a rate of 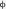 per day and survive for 1/µ days (Eq.5). Zoospore release rates and spore survival were determined experimentally under sterile conditions^28^. These values were comparable to ones reported from close relatives to *S. destruens*^9^. We have yet to develop a method by which to determine spore or zoospore numbers in non-sterile water, so these empirically determined values were used.
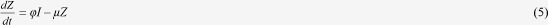


The most appropriate model parameters were determined by establishing a biologically realistic range for parameter values and systematically testing the model output against the observed data for the best fit, using mean squared error as an indicator of model accuracy. To remove any bias from selecting initial values, a 25% random variation was introduced for all parameters for each model iteration. Direct and environmental transmission (β and ε, respectively), and mortality rate due to infection (α) were optimised in R (Version 0.97.551 © 2009-2012) also incorporating random variation for 100,000 iterations, using the optim() function in the *lattice* package^37^. Following the randomised iterations, the model parameters that resulted in the lowest mean squared error were selected. A sensitivity analysis was performed by independently varying each parameter until it resulted in a significant change in mean squared error, thus establishing a range of possible values for each parameter.

To examine the role of direct transmission in crowded environments β was set to 0 to observe how the epidemic would progress.

## Results

### Model parameterization

**Figure 3:**
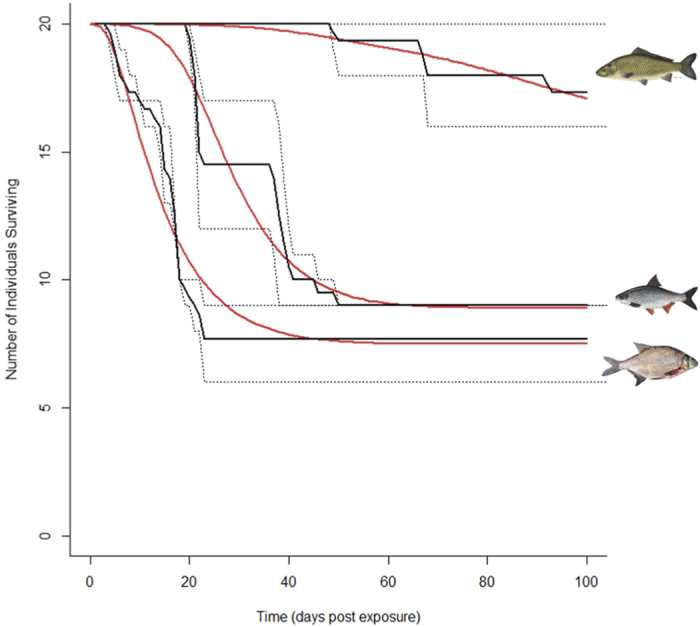
The model output for an outbreak of *Sphaerothecum destruens* in juvenile bream *Abramis brama,* roach *Rutilus rutilus* and carp *Cyprinus carpio*. The model output (red) is compared with the observed data (black) from Andreou *et al* (2012) for *Abramis brama* (lower)*, Rutilus rutilus* (middle) and *Cyrpinus carpio* (top). The minimum and maximum values of the three replicate samples are shown as dashed lines. Images are all in the public domain as copyrights have expired: http://commons.wikimedia.org/wiki/File:Braxen,_Iduns_kokbok.jpg, http://commons.wikimedia.org/wiki/File:Rutilus_rutilus5.jpg, http://commons.wikimedia.org/wiki/File:Cyprinus_carpio3.jpg

Infections in bream were rapid with a minimum length of 10 days of infection from first exposure and an epidemic occurring within 7 days (Fig. 3). Bream have higher transmission rates (β = 0.15-0.99, ε = 0.12-0.7) than the other host species. This is supported by the results of experimental infections, where bream were highly susceptible to *S. destruens* infection and represented the most sensitive host. During these experimental challenges to the pathogen, all mortalities occurred within 23 days of the last exposure to the pathogen, suggesting both a short incubation rate and high transmission rate between infected hosts.

Although the infection rate was lower than for bream, roach are also highly susceptible to infection with *S. destruens*, with a minimum infection time of 26 days after the first exposure to the first mortality (Fig. 3). Transmission parameters for roach optimised to 0.08-0.1 (β), while environmental transmission was much lower at 0.003-0.007. In contrast, carp are not as sensitive to infection as the other species, despite being tested at the same life stage (Fig. 3). They experienced a significantly longer timescale of infection, with the first mortality occurring 55 days post first exposure to *S. destruens* spores. Overall, carp appeared to be more resistant to the pathogen (only 8% of the treatment group experienced mortality and survivors had no *S. destruens* DNA detected 11 months post exposure), indicating a full recovery or a natural immunity to the pathogen. The parameterization reflected these observations, with direct and indirect transmission values at 0.0155-0.017 and 0.0008-0.0012 respectively. Mortality from infection (α = 0.017-0.025) is significantly lower than that of bream and roach.

**Figure 4:**
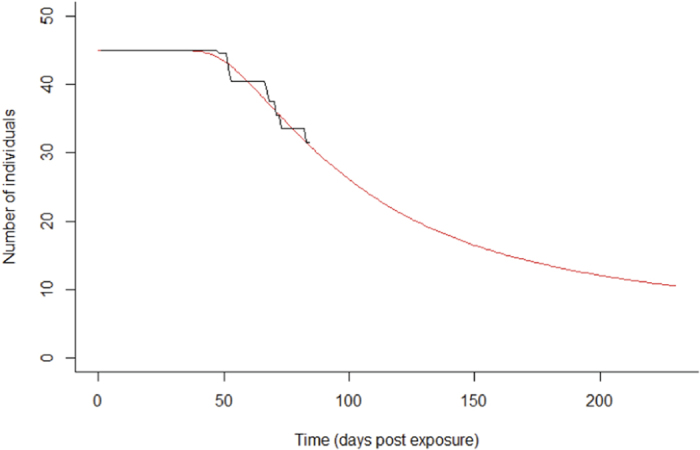
Surviving population of *Leucaspius delineatus* when exposed to *Sphaerothecum destruens*. The model output (red) has been fitted to Paley *et al* (2012) published data (black) and projected for 250 days, to observe how the epidemic would progress.

In sunbleak, the outcome of the model was sensitive to other parameters, as a result of the different experimental setup. While both incubation (0.23-0.233) and recovery rate (0.14-0.17) were still crucial determining factors of the infection, parameters related to spore release including threshold levels for each stage of infection were also important. This demonstrates why it was necessary to include saturation functions for each parameter (Fig. 4). All parameter ranges are listed in Table 2.

### Sensitivity analysis

**Figure 5:**
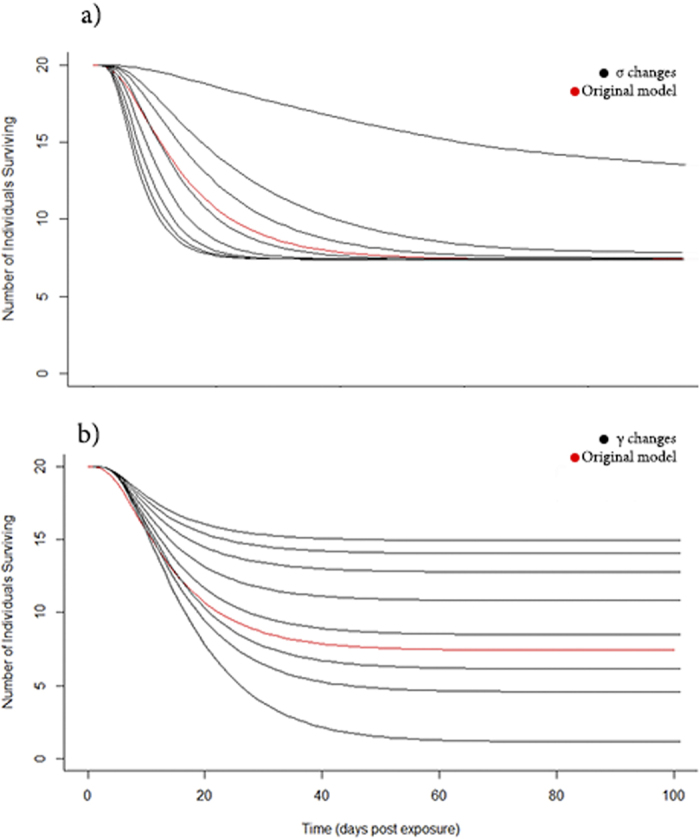
Sensitivity analysis of incubation rate and recovery parameters. Changing the parameter values of σ (incubation) (**a**) and γ (recovery) (**b**) from the original common bream *Abramis brama* model values of 0.1 (shown in red) reveals how largely they affect the outcome of the *Sphaerothecum destruens* epidemic. For σ, values lower than 0.1 (shown are 0.01, 0.05, 0.075) indicating a longer time of incubation, tend to slow down the epidemic curve, although the same number of individuals eventually succumb to the disease. Higher values of σ (0.125, 0.2, 0.3, 0.4, 0.5) speed up the progression of the epidemic, however the epidemics plateau at the same level as other values. In contrast, lower values of γ (tested at the same levels as σ) from 0.1 (indicating longer time to recovery, here shown under the model values in red) severely reduced population survival.

**Figure 6:**
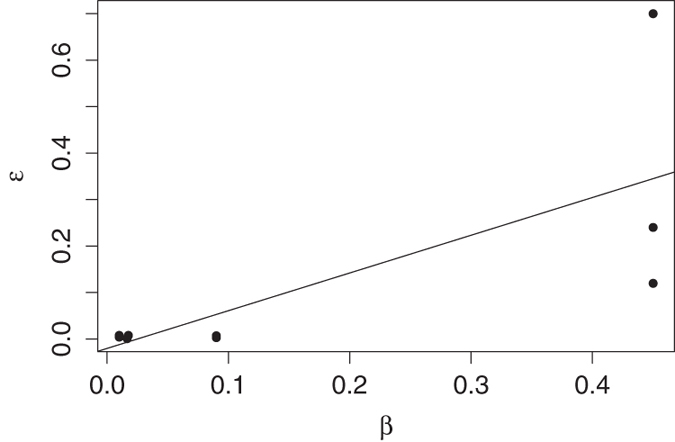
A comparison of direct and environmental transmission values for different species. The minimum, optimised, and maximum values for environmental transmission are plotted against the optimised direct transmission for each species. Initially it appears as though there is a significant linear relationship between direct and environmental transmission (lm: ε = 0.811β – 0.02016, p < 0.001). However, when the leverage of the residuals is examined, it is clear that this relationship is only caused by the significantly higher transmission values for bream. When these are excluded, there is no linearity observed.

The outcome of the model was most sensitive to the rate of recovery (γ) (alternatively duration of infection 1/γ). The incubation rate of the pathogen (σ) also greatly affected the duration of an epidemic (Fig. 5). The rate of mortality from infection (α) was important in the progression of an epidemic in bream and roach, the two most susceptible species. When direct transmission was set to 0, environmental transmission increased. However, there is no collinearity between direct and environmental transmission (Fig. 6), and this increase is largely due to the confines of the data and model structure. Direct and environmental transmission operate at different timescales so are independent of one another; while direct transmission requires contact between infected individuals, environmental transmission occurs on a longer timescale that is dependent on the lifetime of infectious propagules. Transmission is more dependent on population density, although to different degrees for direct and environmental transmission. Thus, the optimised levels of environmental transmission are not sufficient to cause the observed outbreaks in every species, and direct transmission is necessary. It was expected that direct transmission plays an important role in these conditions, and this result supports that.

Other parameters only substantially affected the model output when they were altered by magnitudes of 10-1000. For parameters that did not affect the model output except when altered by a magnitude of ≥100, we set a range of values that were biologically relevant for this particular pathogen, based on our existing empirical knowledge.

## Discussion

Direct transmission through contact between infectious and susceptible individuals (such as in farming conditions or breeding aggregations) was predicted to play a stronger role in the development of the epidemic. Our results have also identified that the incubation rate (σ) and length of infection (γ) are the two key parameters influencing the severity of *S. destruens* outbreaks. Altering these parameters greatly affected transmission and thus the progression of the epidemic^38^ with the incubation rate largely impacting the pace of the epidemic. At the community level, species with a long infection time that are also tolerant to the parasite could act as reservoirs by shedding low levels of infectious spores over a longer time period, potentially leading to a sustained disease outbreak. This has a direct implication in the management of future fungal outbreaks.

Fungal spores can survive in the environment outside their hosts for several days, increasing the probability of contact with sensitive hosts^28^. For example, the saprobic free-living stages of the chytrid fungus have been shown to be a main driver of epidemics of this pathogen^9^. The documented variation in the number and longevity of *S. destruens*’ spores and zoospores may have played a minimal role in driving the epidemic in these closed systems, as the experimental numbers used were well above infection threshold levels. This variation however could play a more significant role for environmental transmission in natural systems where host densities and water flow vary. The high levels of transmission and susceptibility observed here confirm the risk posed by *S. destruens* of spreading to new aquatic areas. This is further reinforced by the existence of a reservoir host that has a broad thermal tolerance and can disperse rapidly^28^,^30^. Currently, bream is both the most sensitive to and the highest transmitter of the disease, which may affect communities to varying degrees based on their structure^3^,^39^. As a generalist parasite, *S. destruens* shows a range of infectivity of hosts and an increased species richness can in effect lower the prevalence of the disease in that environment^3^. Determining which community composition can best constrain pathogen emergence and prevent a widespread outbreak has important implications for controlling disease risk and ultimately for species conservation.

Ecosystems are rapidly changing and will continue to do so as a result of global changes such as significant alterations in land-use, species introductions and habitat destruction^8^,^40^. These all directly or indirectly affect the environmental transmission and contact frequencies between potential hosts, with a knock-on effect on the evolutionary bi-stability of the pathogen’s transmission patterns^11^,^41^,^42^. Although creating this evidence-based model has established an important baseline for understanding disease dynamics in fungal parasites, a more comprehensive community level network model should be developed in order to really understand the dynamics and potential impacts of such pathogens on host populations and biodiversity. The role of host community structure in disease dynamics, in particular spore and zoospore production, will alter the incubation rate and length of infection modulating the severities of observed infections and needs to be further explored.

## Author Contributions

Conceived the study: R.E.G., D.A., F.N.A., Conceived and designed the cohabitation and zoospore experiments: R.E.G., D.A. Performed the cohabitation and zoospore experiments: D.A. Analysed the data: F.N.A., B.R., R.E.G. Wrote the paper: F.N.A., D.A., R.E.G., J.R.B., B.R.

## Additional Information

**How to cite this article**: Al-Shorbaji, F. N. *et al.* The alternate role of direct and environmental transmission in fungal infectious disease in wildlife: threats for biodiversity conservation. *Sci. Rep.* **5**, 10368; doi: 10.1038/srep10368 (2015).

## Supplementary Material

Supplementary Information

## Figures and Tables

**Table 1:** Akaike’s information criterion (AIC) for models that include or exclude parameter saturation functions.

**Saturation function included for:**	**AIC**			
	***Abramis brama***	***Rutilus rutilus***	***Cyprinus carpio***	***Leucaspius delineatus***
ε	431.544 (MSE=63.78)	433.19 (MSE=64.84)	226.29 (MSE=8.19)	No convergence
ε, σ	433.06 (MSE=63.47)	435.44 (MSE=65)	237.27 (MSE=8.96)	363.99 (MSE=61.50)
ε, σ ,γ	435.56 (MSE=63.79)	437.13 (MSE=64.80)	229.06 (MSE=8.09)	367.59 (MSE=62.68)
ε, σ, γ, α	436.73 (MSE=63.26)	439.75 (MSE=65.20)	237.29 (MSE=8.61)	362.07(MSE=57.31)

This shows that saturation functions are needed for all parameters in the course of infection for *Leucaspius delineatus* (dAIC 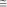 2) while a saturation function is required only for infection for the other host species. To create a model that fits all species, the model that included saturation functions for all parameters was chosen.

**Table 2:** Parameter ranges for the model, with definitions and parameter ranges considered in the model’s construction.

**parameter**	**definition**	**estimated value**	**range considered**	**reference**
β	Direct transmission	0.15-0.99 *(A. brama*) 0.08-0.1 (*R. rutilus*) 0.0155-0.017 (*C. carpio*) 0.015- 0.02 (*L. delineatus*)	0.0001-0.999	This work
ε	Environmental (indirect) transmission	0.12-0.7 *(A. brama*) 0.003-0.007 (*R. rutilus*) 0.0008-0.0012 (*C. carpio*) 0.0074-0.008 (*L. delineatus*)	0.0001-0.999	This work
α	Mortality from infection	0.165-0.18 *(A. brama*) 0.129-0.133 (*R. rutilus*) 0.017-0.025(*C. carpio*) 0.22-0.3 (*L. delineatus*)	0.0001-0.999	This work
γ	Rate of recovery	0.095-0.105(*A. brama*) 0.099-0.101(*R. rutilus*) 0.065-0.072 (*C. carpio*) 0.14-0.17 (*L. delineatus*)	10-25 days	This work
σ	Incubation rate	(*A. brama*) 0.095-0.11(*R. rutilus*) 0.013-0.0805 (*C. carpio*) 0.23-0.233 *(L. delineatus*)	5-15 days	This work, Andreou *et al.* (2012)
f	Rate of the release of spores from infected host	0-10000 sporesday ^−1^ (*A. brama*) 0-4000 spores day ^−1^ (*R. rutilus*) 350-650 spores day ^−1^ (*C. carpio*) 330-350 spores day ^−1^(*L. delineatus*)	50-10000 spores day ^−1^	This work, Andreou (personal communication)
μ	Mortality rate of spores and zoospores	0.2 (5 day lifespan)	1-8 days	Andreou *et al.* (2009)
