# Supplementary material for: The alternate role of direct and environmental transmission in fungal infectious disease in wildlife: threats for biodiversity conservation
Source: Sci Rep. 2015 May 20;5:10368. doi: 10.1038/srep10368 (PMC4438611; doi:10.1038/srep10368)
Supplement: Supplementary Information [file srep10368-s1.doc]

**The alternate role of direct and environmental transmission in fungal infectious disease in wildlife: threats for biodiversity conservation**

Farah N. Al-Shorbaji^1*^, Rodolphe E.Gozlan^2^, Benjamin Roche^3^, J. Robert Britton^1^, and Demetra Andreou^1^

**1** Bournemouth University, Fern Barrow, Talbot Campus, Poole, Dorset, BH12 5BB, UK

**2** UMR BOREA IRD-MNHN-Université Pierre et Marie Curie, Muséum National d’Histoire Naturelle, 47 rue Cuvier, 75231 Paris cedex 5, France

**3** International Research Unit UMMISCO, Center for Mathematical and Computational Modeling of Complex Systems, Research Institute for Development (IRD), [32 Avenue Henri Varagnat](x-apple-data-detectors://18), [93143 Bondy Cedex, France](x-apple-data-detectors://18)

*Corresponding author at: [falshorbaji@bournemouth.ac.uk](mailto:falshorbaji@bournemouth.ac.uk)

**Supplementary materials**

Table S1. Known hosts of *Sphaerothecum destruens*, prevalence and details of infection.

| host species | infection details | reference |
| --- | --- | --- |
| Atlantic salmon  *Salmo salar* | Chronic mortality with 75% prevalence, systemic infection, lesions in kidneys and liver | Hedrick et al. (1989) |
| Chinook salmon  *Oncorhynchus tshawytscha* | Over 95% mortality, systemic disease, especially in spleen and kidney | Elston et al. (1986), Harrell et al. (1986) |
| Coho salmon  *Oncorhynchus kisutch* | 98% of experimental population infected, widespread disseminated infection | Arkush et al. (1998) |
| Rainbow trout  *Oncorhynchus mykiss* | 42.5% of experimental population infected, less severe infection than salmon | Arkush et al. (1998) |
| Brown trout  *Salmo trutta* | 43.3% of experimental population infected, less severe infection than salmon | Arkush et al. (1998) |
| Brook trout  *Salvelinus fontinalis* | Only 2.6% of experimental population infected, possible role as a tolerant carrier | Arkush et al. (1998) |
| Sunbleak  *Leucaspius delineatus* | 96% population decline over 3 seasons, total inhibition of spawning | Gozlan et al. (2005) |
| Fathead minnow  *Pimephales promelas* | Loss of condition, 60% mortality over 4 months, inhibition of spawning | Gozlan et al. (2005) |
| Bream  *Abramis brama* | 53% mortality over 23 days, infection found in kidneys, liver, intestines | Andreou et al. (2012) |
| Roach  *Rutilus rutilus* | 37% mortality over 50 days, low prevalence detected in liver, kidneys, intestines | Andreou et al. (2012) |
| Common carp  *Cyprinus carpio* | 8% mortality over 3 months, infection found in intestines | Andreou et al. (2012) |
| Topmouth gudgeon  *Pseudorasbora parva* | Tolerant host | Gozlan et al. (2005) |

**Modelling assumptions**

While our mathematical model relies on the well-established SIR framework, one of our assumptions deserves deeper explanation. We assume pathogen life-history traits are associated with spore levels through saturating functions. This assumption has been made in order to present a general model for this pathogen across different host species and experimental setups.

Some experimental setups infect hosts through bath immersion with millions of spores while other experiments infect hosts through injection. In the case of injection, the level of spores/zoospores in the water at initial time is very low, and as such the saturation functions made a significant difference in model outputs (Figure S1). In bath immersion infections the saturation functions did not make a significant difference to the results due to the high initial levels of spores. However, we chose to include saturation functions for the pathogen life-history traits in order for the model to be applicable to the widest range of scenarios and experimental datasets.

It is worth pointing out that the general model does not suffer from the inclusion of this saturating function. In the case of *S. destruens*, spore levels in the water do not reach 0, even at extreme temperatures^28^. As such there is no risk of the parameter values approaching 0 (Figure S2). This method also has the advantage of minimising the number of constants and parameters that need to be optimised (k values) compared with a logarithmic function. Given the alternative methods used and the results obtained, it is clear that including zoospore saturation is a key component in the generalised model.


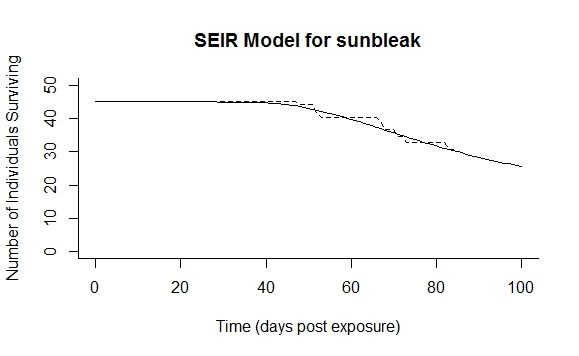


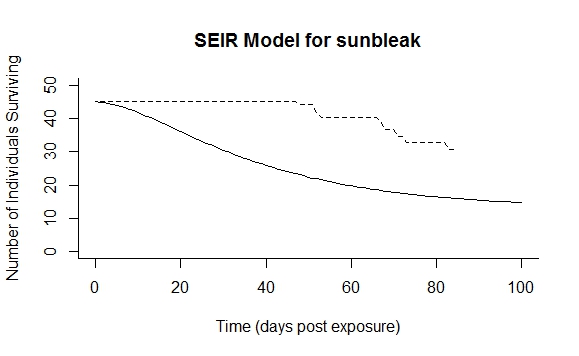


Figure S1. The generalised model including all the saturation functions (upper panel) is the best fit for sunbleak *Leucaspius delineatus* data ^26^(dashed line). When the model only includes saturation for environmental uptake of spores (ε), it does not fit the observed data (lower panel).


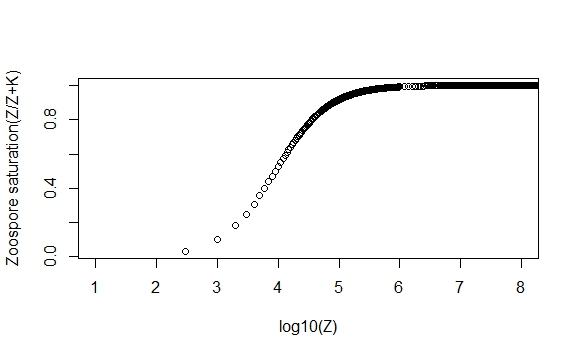


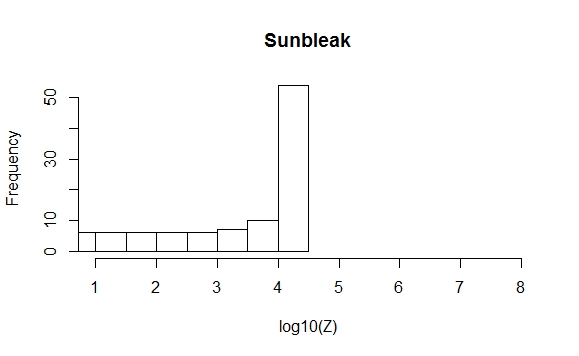


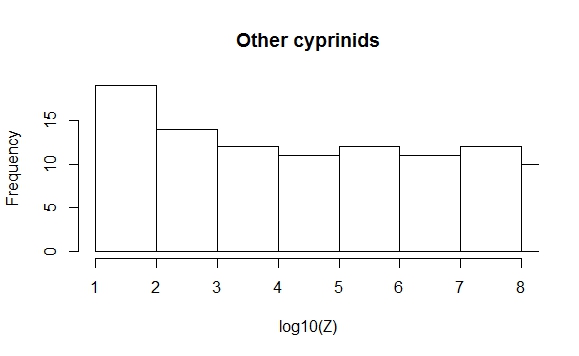


Figure S2. Top panel shows spore and zoospore saturation levels for the model. Middle panel shows sunbleak *Leucaspius delineatus* spore and zoospore levels demonstrating that the saturation function is important to note the differences in disease progression, especially when compared with the experimental set up for bream, carp and roach (lowest panel).
